# Constantine Lippe, A.M., M.D.

**Published:** 1885-01

**Authors:** 


### In Memoriam.

### CONSTANTINE LIPPE, A. M., M. D.

Dr. Constantine Lippe died suddenly January 1st, 1885, at his residence in New York.

We feel sure the announcement will cause universal regret among homœopathists generally that such an able and energetic worker should be so suddenly taken from the profession which he loved and so well adorned. Among patients and friends his loss will be grievously lamented, and his place will be long vacant. Universal sympathy will be extended to his father, Dr. Adolph Lippe, who loses, in two brief weeks, a cherished daughter and a valued son.

Dr. Constantine Lippe was in his forty-fifth year, was a conscientious and able practitioner, a pure homœopathist, a frequent contributor to this and other journals; also the author of a valued repertory, which he was just rewriting for a second edition.

*Requiescat in Pace.*

